# Obesity Class and Severity of Metabolic Emergencies: A Single-Center Retrospective Five-Year Study

**DOI:** 10.3390/healthcare13060617

**Published:** 2025-03-12

**Authors:** Iulia Najette Crintea, Alexandru Cristian Cindrea, Teodor Florin Fulga, Cosmin Iosif Trebuian, Adina Maria Marza, Alina Petrica, Ovidiu Alexandru Mederle, Romulus Timar

**Affiliations:** 1 Department of Surgery, “Victor Babes” University of Medicine and Pharmacy, 300041 Timisoara, Romania; iulia.crintea@umft.ro (I.N.C.); alexandru.cindrea@umft.ro (A.C.C.); marza.adina@umft.ro (A.M.M.);; 2 Emergency Department, Emergency Clinical Municipal Hospital, 300079 Timisoara, Romania; 3 Faculty of Cybernetics, Statistics and Economic Informatics, The Bucharest University of Economic Studies, 010374 Bucharest, Romania; teoflorin02@gmail.com; 4 Department of Anesthesia and Intensive Care, Emergency County Hospital, 320210 Resita, Romania; 5 Emergency Department, “Pius Brinzeu” Emergency Clinical County Hospital, 300736 Timisoara, Romania; 6 “Pius Brinzeu” Emergency County Hospital, 300723 Timisoara, Romania; timar.romulus@umft.ro; 7 Second Department of Internal Medicine, “Victor Babes” University of Medicine and Pharmacy, 300041 Timisoara, Romania

**Keywords:** obesity, metabolic emergencies, emergency department, comorbidities, retrospective study

## Abstract

**Background/Objectives**: This study aims to investigate the impact of obesity severity on the prevalence and outcomes of acute metabolic emergencies in the emergency department (ED) setting, with a specific focus on obesity class stratification and associated metabolic complications. **Methods**: This retrospective, single-center study analyzed data from 433 patients admitted to the ED of the Timisoara Municipal Emergency Hospital between January 2019 and March 2024. Patients were classified according to WHO obesity grades (Class I: BMI 30.0–34.9 kg/m^2^, Class II: 35.0–39.9 kg/m^2^, Class III: ≥ 40.0 kg/m^2^). The prevalence and severity of metabolic emergencies, including hyperglycemic crises, acute kidney injury (AKI), and severe electrolyte imbalances, were compared across obesity classes. **Results**: Obese patients (37.2%) exhibited a significantly higher prevalence of metabolic emergencies than non-obese individuals (*p* < 0.001). Hyperglycemia was present in 27.9% of obese patients vs. 11.0% of non-obese patients (*p* < 0.001). AKI incidence nearly doubled in obese patients (12.4% vs. 5.5%, *p* = 0.01). Logistic regression identified Class III obesity as an independent risk factor for metabolic emergencies (adjusted OR = 3.2, 95% CI: 2.1–4.9, *p* < 0.001). **Conclusions**: The severity of metabolic emergencies increases with increasing obesity class, emphasizing the need for obesity-specific risk stratification in ED settings. Routine monitoring of metabolic markers and early intervention strategies should be prioritized for high-risk obese patients.

## 1. Introduction

Obesity is a major global health concern, contributing to both chronic and acute medical conditions. According to the WHO, obesity rates have tripled since 1975, affecting over 650 million adults [1]. This trend is particularly concerning in emergency department (ED) settings, where obesity can significantly complicate patient care and influence clinical outcomes.

In Romania, obesity prevalence has increased significantly, affecting emergency healthcare services and resource allocation [2,3]. Data on how obesity severity correlates with the frequency and type of acute metabolic emergencies in the Romanian emergency healthcare context remain scarce. Understanding these relationships is crucial for developing targeted interventions and informing public health policies to mitigate the impact of obesity on healthcare resources.

Obesity severity is classified according to the WHO criteria into three grades based on BMI, which allows for risk stratification. This standardized classification allows for a more precise analysis of obesity severity in relation to acute metabolic crises, providing valuable insights into the risk stratification of associated health complications, while also guiding early intervention strategies [4,5].

Obesity contributes to metabolic emergencies through mechanisms such as chronic inflammation, insulin resistance, and electrolyte imbalances, increasing the risk of acute conditions in the emergency department [6]. In the ED setting, patients with obesity often present with these metabolic crises, which can escalate quickly if not managed promptly [7].

The link between obesity and metabolic markers is critical in emergency care. Obesity disrupts key parameters, notably electrolyte balance, with insulin resistance and hormonal changes increasing the risk of both hyper- and hypokalemia in acute illness [8]. These alterations can precipitate or worsen cardiac arrhythmia, requiring more careful ED monitoring. Sodium metabolism is similarly disrupted through changes in the renin–angiotensin–aldosterone system, affecting fluid balance and blood pressure regulation [9]. This disruption can complicate the management of acute conditions, particularly in patients with concurrent heart failure or hypertension.

Renal function in obesity is often compromised, with altered creatinine and urea levels reflecting metabolic and circulatory disturbances [10]. Additionally, changes in albumin, calcium, and inflammatory markers (CRP, procalcitonin) can affect clinical assessment and complicate emergency management [11,12].

These metabolic alterations have significant clinical implications. Emergency physicians must adjust laboratory interpretations and drug dosing based on obesity and metabolic status. Obesity also increases the risk of comorbidities like type 2 diabetes, hypertension, and cardiovascular disease [13]. These comorbid conditions contribute to the complexity of patient management and seemingly increase the likelihood of metabolic instability [14], with certain metabolic derangements being potentially predictive of an increased risk for complications during emergency care [15]. In fact, the combination of obesity and associated comorbidities not only exacerbates the severity of acute episodes but also leads to longer hospital stays and higher rates of intensive care unit (ICU) admissions [16]. Moreover, the presence of certain metabolic derangements may predict an increased risk for complications during emergency care, such as the need for intensive care admission or prolonged hospital stays [15]. Understanding these relationships is, therefore, essential for risk stratification and early intervention in the emergency department setting.

Although obesity is well known to contribute to chronic diseases, its role in acute metabolic emergencies remains underexplored [17]. This study aims to bridge this knowledge gap by analyzing the impact of obesity severity on metabolic complications in a large emergency department in Romania. This retrospective study analyzes demographics, comorbidities, and laboratory findings to assess the link between obesity severity and acute metabolic emergencies in the ED. It aims to evaluate obesity classes in risk stratification, laboratory alterations, and emergency management. Understanding these associations can aid in identifying high-risk patients, optimizing ED resource allocation, and developing obesity-specific protocols. Given rising obesity rates and their impact on emergency care [18,19], these findings may guide preventive strategies and targeted interventions.

## 2. Materials and Methods

Study Design and Setting. This retrospective observational study was conducted in the ED of the Timisoara Municipal Emergency Hospital. The study was conducted between 1 January 2019 and 1 March 2024. All patient data collected during this period were included in the final analysis. Data processing and statistical analysis were completed after March 2024, ensuring that the entire dataset was utilized for the results presented in this manuscript. The study protocol was approved by the Institutional Ethics Committee of the Timisoara Municipal Emergency Hospital (approval number: E-5945/05.11.2024) and conducted in accordance with the Declaration of Helsinki. Written informed consent was obtained from all participants or their legal representatives at the time of hospital admission for the use of their anonymized data in future research.

**Figure 1:**
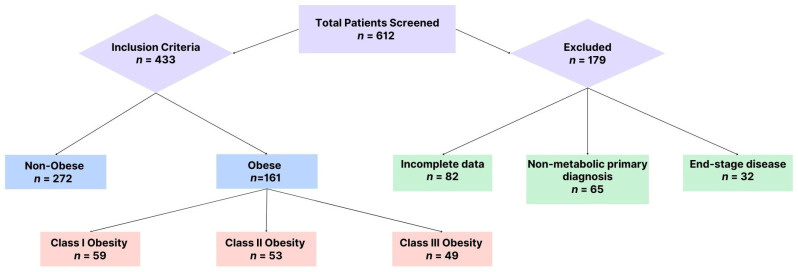
Patient selection.

Study Population. Initially, a total of 612 cases were screened, of which only 433 patients who presented to the ED with symptoms indicative of acute metabolic emergencies, such as electrolyte imbalances, hyperglycemia, or acute kidney injury, met the inclusion criteria and were analyzed further (see Figure 1).

Patients aged 18 years or older who presented directly to the emergency department (ED) with metabolic emergencies and required hospital admission for further management were eligible for inclusion. To ensure comprehensive data analysis, only cases with complete clinical records, including demographic characteristics, admission and discharge dates, vital signs, relevant comorbidities, and laboratory test results (such as glycemia, serum potassium, serum sodium, serum creatinine, and urea), were included in the study.

Metabolic emergencies were classified into three major categories: hyperglycemic crises, severe electrolyte disturbances, and acute kidney injury (AKI). Hyperglycemic crises encompassed diabetic ketoacidosis (DKA) and hyperosmolar hyperglycemic state (HHS), defined by specific diagnostic criteria. DKA was characterized by blood glucose levels exceeding 250 mg/dL, an arterial pH below 7.3, and the presence of urinary ketones. In contrast, HHS was identified in patients with blood glucose levels above 600 mg/dL and serum osmolality greater than 320 mOsm/kg. Severe electrolyte imbalances were determined based on potassium and sodium levels, with hyperkalemia defined as potassium concentrations above 6.0 mmol/L and hypokalemia as values below 2.5 mmol/L. Sodium imbalances were considered severe when serum sodium levels dropped below 125 mmol/L or exceeded 155 mmol/L, particularly in cases presenting with associated symptoms or electrocardiographic abnormalities. AKI was diagnosed according to the KDIGO classification, with Stage 2 AKI identified by a serum creatinine increase of 2.0–2.9 times the baseline or a urine output reduction below 0.5 mL/kg/h for at least 12 h. Stage 3 AKI was defined by a creatinine increase of at least 4.0 mg/dL, a threefold rise from baseline levels, or a urine output decline to below 0.3 mL/kg/h for more than 24 h. The initiation of renal replacement therapy was also a defining criterion for severe AKI.

Certain patients were excluded from the study based on predefined criteria. Individuals younger than 18 years old or those with incomplete medical records—such as missing key laboratory results or admission and discharge details—were not considered. Cases where the primary reason for admission was unrelated to metabolic emergencies, including trauma-related conditions (e.g., fractures, appendicitis), neurological emergencies without metabolic involvement (e.g., stroke, seizures), or cardiovascular events without electrolyte disturbances (e.g., myocardial infarction without metabolic abnormalities), were also excluded. Additionally, patients with COVID-19 were removed from the dataset, as their metabolic disturbances might have been influenced by SARS-CoV-2 infection. Metabolic imbalances secondary to non-metabolic conditions, such as major surgical procedures, cancer treatments, or end-stage chronic kidney disease, were not included in the final analysis. Finally, patients receiving only palliative or comfort care, where active metabolic management was not pursued, were excluded to maintain consistency in the evaluation of acute metabolic emergencies.

Data Collection. We retrospectively gathered data from electronic medical records using a standardized data collection form, which included demographics (age, sex), clinical comorbidities (e.g., diabetes, hypertension, chronic kidney disease), and laboratory findings. Key laboratory parameters collected were blood glucose, serum electrolytes (potassium, sodium), and renal function markers (urea, creatinine). These variables were selected to assess the metabolic disturbances associated with obesity and their impact on acute clinical presentations. The primary focus was on assessing obesity and its association with metabolic emergencies. Height and weight were measured using calibrated equipment during ED admission, with BMI calculated as weight (kg)/[height (m)]^2^. Obesity was defined according to the WHO criteria based on BMI, with patients classified as obese if their BMI was ≥ 30 kg/m^2^. Obesity severity was further categorized into Class I (BMI 30.0–34.9 kg/m^2^), Class II (BMI 35.0–39.9 kg/m^2^), and Class III (BMI ≥ 40.0 kg/m^2^) [20]. Key metabolic markers linked to obesity, such as blood glucose and electrolyte levels, were emphasized. Data quality was ensured through the following: double data entry by two independent researchers; regular consistency checks; resolution of discrepancies through source document verification; and handling of missing data through multiple imputations when appropriate. Primary outcomes included the following: (1) prevalence of metabolic emergencies across obesity classes; (2) severity of laboratory modifications; and (3) emergency department outcomes. Secondary outcomes comprised the following: (1) length of ED stay; (2) need for ICU admission; and (3) in-hospital mortality.

Statistical Analysis. Statistical analysis was conducted using MedCalc^®^ Statistical Software version 20.118 (MedCalc Software Ltd., Ostend, Belgium; 2022). The sample size was calculated to detect a 20% difference in the prevalence of metabolic emergencies between obese and non-obese groups, with 80% power and α = 0.05. Prior to analysis, data normality was assessed using the Shapiro–Wilk test. Descriptive statistics were utilized to summarize the study population’s characteristics, with continuous variables presented as means with standard deviations for normally distributed data or medians with interquartile ranges for non-normally distributed data. Categorical variables were presented as counts and percentages. Comparisons between obese and non-obese groups were performed using independent *t*-tests for normally distributed continuous variables, or Mann–Whitney U tests for non-normally distributed data. Chi-square tests were used for categorical variables, with Fisher’s exact test applied when expected cell counts were less than five. For comparisons across obesity classes, one-way ANOVA or Kruskal–Wallis tests were used based on data distribution. Post hoc analyses were conducted using Tukey’s HSD test for ANOVA or Dunn’s test with Bonferroni correction for Kruskal–Wallis. Spearman’s rank correlation coefficient was calculated to assess relationships between BMI and continuous variables. Multivariable logistic regression analysis was performed to evaluate the association between obesity class and metabolic emergencies, adjusting for age, gender, and comorbidities. Statistical significance was set at *p* < 0.05, and all tests were two-tailed. Effect sizes were calculated using Cohen’s d for continuous variables and Cramer’s V for categorical variables.

## 3. Results

From January 2019 to March 2024, 433 patients meeting the metabolic emergency criteria were included in the analysis. The cohort comprised 161 obese patients (37.2%) and 272 non-obese patients (62.8%). Initial analysis of data distribution using the Shapiro–Wilk tests revealed that age (W = 0.982, *p* = 0.324), BMI (W = 0.978, *p* = 0.287), blood glucose (W = 0.975, *p* = 0.263), and electrolyte parameters (potassium: W = 0.981, *p* = 0.312; sodium: W = 0.979, *p* = 0.298) followed normal distributions. However, renal function markers showed non-normal distributions (urea: W = 0.892, *p* < 0.001; creatinine: W = 0.901, *p* < 0.001). These findings guided our selection of appropriate statistical tests for subsequent analyses.

The study categorized obese patients (n = 161) into three classes based on BMI criteria. The distribution revealed that Class I obesity was the most prevalent, accounting for 36.6% of the cohort (n = 59), followed closely by Class II with 33.3% (n = 53), whereas Class III obesity represented 30.5% (n = 49). A chi-square test was conducted to assess the statistical significance of the distribution of obesity grades compared to an expected uniform distribution. The analysis yielded a chi-square statistic of 50.76 with three degrees of freedom, and a *p*-value of < 0.001, indicating a highly significant deviation from the expected uniform distribution.

In Table 1, obese patients had a significantly higher mean age (72.2 ± 9.4 years) compared to non-obese patients (68.1 ± 10.1 years, *p* = 0.03). Gender distribution showed a slightly higher proportion of females among obese patients (55%) compared to non-obese patients (48%), though this difference was not statistically significant (*p* = 0.08). Obese patients exhibited a markedly higher BMI (36.3 ± 4.7 kg/m^2^) than non-obese patients (24.5 ± 3.2 kg/m^2^, *p* = 0.001). Comorbidities were notably more prevalent in the obese group. Hypertension was present in 90.7% of obese patients versus 73.5% in non-obese patients (*p* = 0.002). Cardiac disease was slightly less frequent in obese patients (89.4%) compared to non-obese patients (92.3%, *p* = 0.04), while diabetes mellitus showed a higher prevalence in obese patients (15.5%) than in non-obese patients (11.8%, *p* = 0.05). Chronic kidney disease was observed at similar rates in both groups (54.7% vs. 53.3%, *p* = 0.45). The prevalence of COPD was low in both obese and non-obese groups (1.2% vs. 1.5%, *p* = 0.55), and neoplasms were slightly less frequent among obese patients (14.9%) compared to non-obese patients (19.9%, *p* = 0.12). These findings highlight significant associations between obesity and several comorbid conditions, emphasizing the higher clinical burden faced by obese individuals.

In addition to comparing obese and non-obese patients, we performed post hoc comparisons between obesity classes (Class I, Class II, and Class III) to identify significant metabolic and clinical differences across severity levels. Statistical significance for these comparisons was assessed using Tukey’s HSD test for continuous variables and Bonferroni-corrected chi-square tests for categorical variables. The results, summarized in Table 1, indicate a progressive increase in BMI, blood glucose, and creatinine levels from Class I to Class III, with statistically significant differences between Class III and Class I (*p* < 0.001) and between Class III and Class II (*p* < 0.01). Similarly, the prevalence of hypertension and diabetes mellitus was significantly higher in Class III compared to Class I (*p* = 0.001 and *p* = 0.03, respectively). However, no statistically significant differences were observed between Class II and Class III for cardiac disease (*p* = 0.40) or chronic kidney disease (*p* = 0.25), suggesting that these conditions may plateau at higher obesity levels. These findings confirm a dose-response relationship between obesity severity and metabolic derangements, further supporting the need for stratified risk assessment and targeted clinical interventions for Class III obesity patients.

The laboratory findings revealed notable trends across the three obesity classes, as shown in Table 2. Blood glucose levels exhibited a progressive increase from Class I (142.5 ± 12.4 mg/dL) to Class III (168.4 ± 17.5 mg/dL), indicating worsening glucose metabolism with increasing obesity severity. Potassium levels rose modestly across the grades, from 4.3 ± 0.2 mmol/L in Class I to 4.7 ± 0.4 mmol/L in Class III, reflecting a gradual development of electrolyte imbalances commonly associated with Class III obesity. Sodium levels also showed an upward trend, with mean values ranging from 139.5 ± 3.5 mmol/L in Class I to 141.3 ± 3.1 mmol/L in Class III, suggesting potential fluid balance disturbances in individuals with higher obesity grades. Urea levels increased steadily from 44.3 ± 5.2 mg/dL in Class I to 50.1 ± 6.7 mg/dL in Class III, which may indicate declining renal function. Similarly, creatinine levels demonstrated a rising pattern, from 1.3 ± 0.1 mg/dL in Class I to 1.6 ± 0.3 mg/dL in Class III, further supporting the hypothesis of worsening renal function as obesity severity increases.

To evaluate the statistical significance of differences in laboratory findings across obesity grades, one-way ANOVA was performed for each parameter. The analysis for blood glucose revealed a highly significant difference across the three obesity classes (*p* < 0.001), indicating a clear trend of worsening glycemic control as obesity severity increased. Potassium levels also differed significantly (*p* = 0.03), suggesting progressive disruptions in electrolyte balance, while sodium levels exhibited a similarly significant upward trend (*p* = 0.02) with increasing obesity grades, highlighting fluid and electrolyte disturbances.

Renal function parameters showed marked differences across the grades. Urea levels were significantly elevated (*p* = 0.001) as obesity increased, reflecting a possible decline in renal clearance or higher metabolic stress in more obese individuals. Creatinine levels also demonstrated a significant upward trend (*p* = 0.002), further supporting the hypothesis of progressive renal impairment in patients with higher obesity grades.

In Table 3, BMI demonstrated significant correlations with all metabolic parameters, with the strength of association varying across measurements. Blood glucose showed the strongest positive correlation with BMI (rs = 0.52, 95% CI: 0.45–0.58, *p* < 0.001), followed by creatinine (rs = 0.47, 95% CI: 0.40–0.53, *p* < 0.001) and urea (rs = 0.43, 95% CI: 0.36–0.49, *p* = 0.001). Electrolyte parameters demonstrated weaker but still significant correlations, with potassium (rs = 0.31, 95% CI: 0.23–0.38, *p* = 0.03) and sodium (rs = 0.28, 95% CI: 0.20–0.35, *p* = 0.04) both showing positive associations with increasing BMI. These correlations remained significant after adjusting for age, gender, and comorbidities through partial correlation analysis.

**Figure 2:**
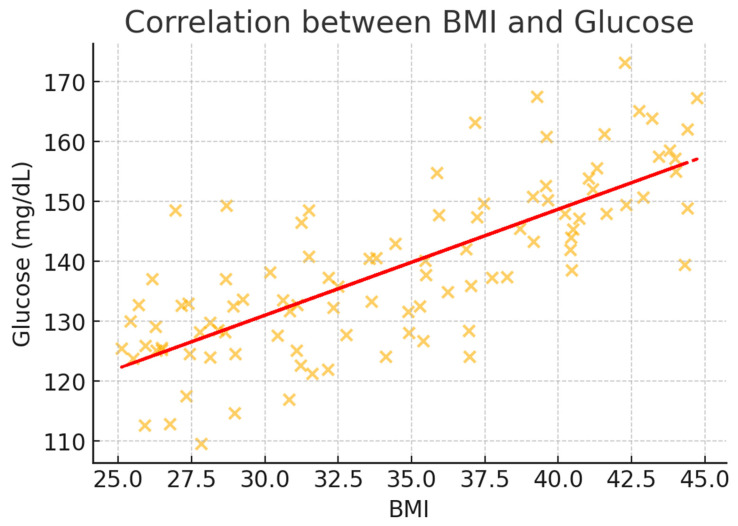
Scatter plot illustrating the correlation between BMI and glucose levels (mg/dL). A positive association is observed, indicating higher glucose levels with increasing BMI. The regression line highlights the upward trend.

**Figure 3:**
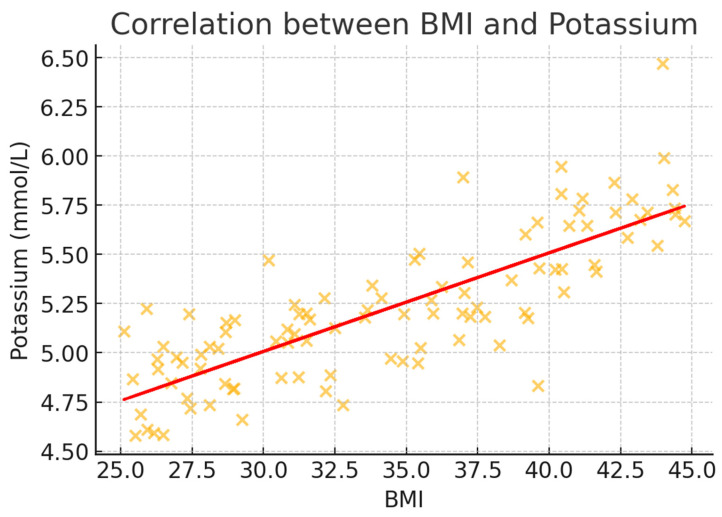
Scatter plot illustrating the correlation between BMI and potassium levels (mmol/L). A weak positive correlation is noted, suggesting a slight increase in potassium levels with higher BMI.

**Figure 4:**
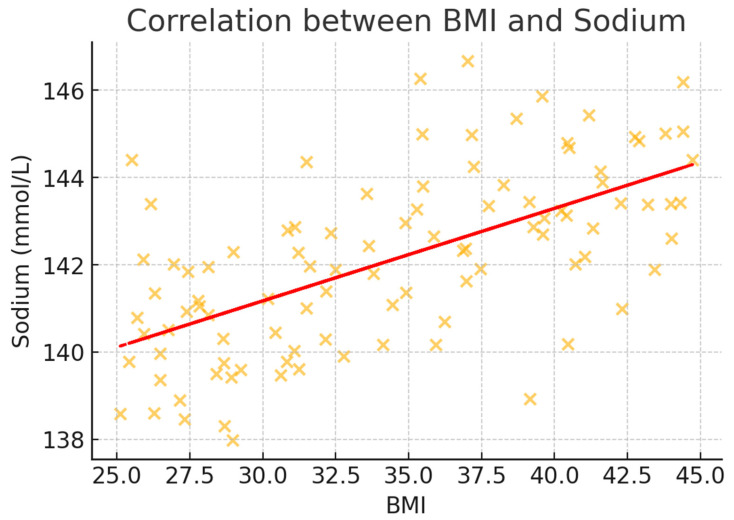
Scatter plot illustrating the correlation between BMI and sodium levels (mmol/L). The data show a weak but positive association, suggesting minor sodium level fluctuations with obesity severity.

**Figure 5:**
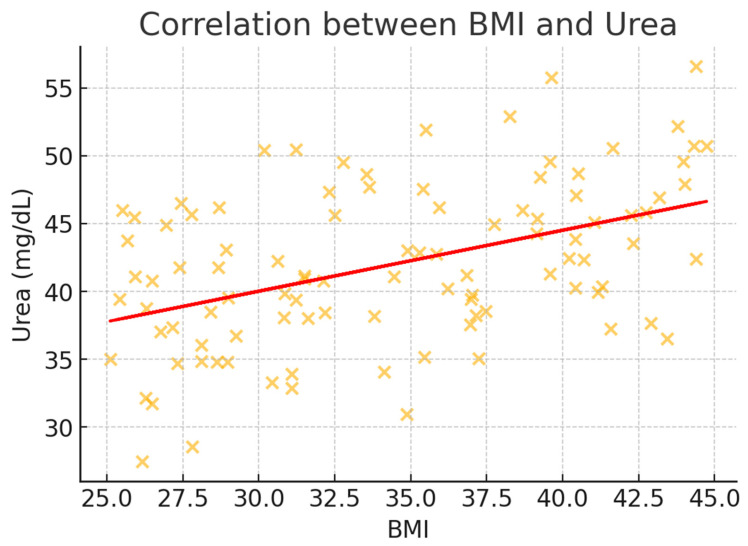
Scatter plot illustrating the correlation between BMI and urea levels (mg/dL). A moderate positive correlation is evident, demonstrating an increase in urea levels with rising BMI, likely reflecting altered renal function in obese individuals.

**Figure 6:**
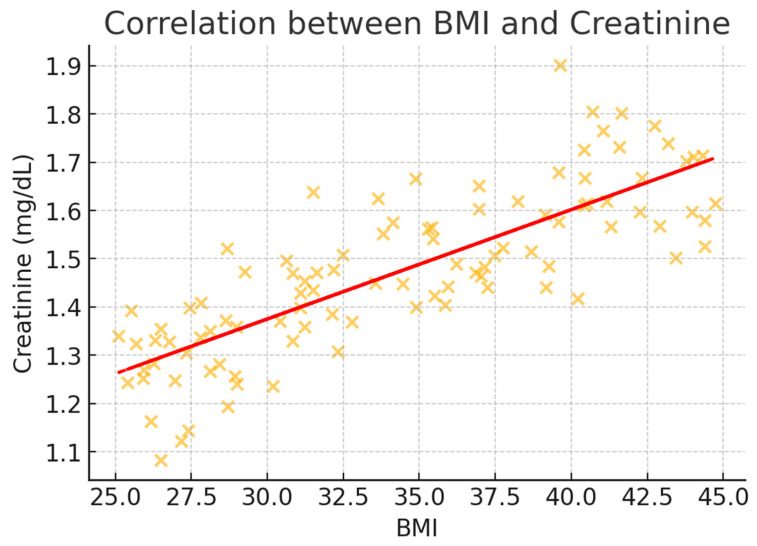
Scatter plot illustrating the correlation between BMI and creatinine levels (mg/dL). A moderate correlation is observed, indicating that higher BMI is associated with increased creatinine levels, which may reflect obesity-related renal stress.

To better illustrate the correlation trends, scatter plots were generated for BMI and key metabolic markers (Figure 2, Figure 3, Figure 4, Figure 5 and Figure 6). These visualizations confirm the positive association between BMI and glucose, creatinine, and urea levels while showing weaker correlations with potassium and sodium. The regression lines further support these findings, demonstrating an increasing trend of metabolic disturbances with obesity severity.

Multivariate logistic regression revealed obesity class as an independent predictor of metabolic emergencies (see Table 4). After adjusting for demographic factors and comorbidities, the risk increased progressively with obesity severity. Compared to non-obese patients, Class I obesity showed an adjusted odds ratio (aOR) of 1.8 (95% CI: 1.2–2.7, *p* = 0.008), Class II demonstrated an aOR of 2.4 (95% CI: 1.6–3.6, *p* < 0.001), and Class III presented the highest risk with an aOR of 3.2 (95% CI: 2.1–4.9, *p* < 0.001). Among comorbidities, diabetes mellitus emerged as the strongest independent predictor (aOR: 2.1, 95% CI: 1.4–3.1, *p* < 0.001), followed by hypertension (aOR: 1.6, 95% CI: 1.1–2.3, *p* = 0.02). Age showed a modest but significant association (aOR: 1.02 per year, 95% CI: 1.01–1.04, *p* = 0.03).

The magnitude of obesity’s impact varied across different parameters, as quantified by standardized effect sizes. Comparing Class III obesity to non-obese patients revealed large effect sizes for BMI (Cohen’s d = 2.8, 95% CI: 2.4–3.2) and blood glucose (d = 1.2, 95% CI: 0.9–1.5). Medium effect sizes were observed for renal function parameters, including creatinine (d = 0.76, 95% CI: 0.52–1.00) and urea (d = 0.68, 95% CI: 0.44–0.92). For categorical outcomes, obesity showed moderate associations with metabolic emergencies (Cramer’s V = 0.29) and hypertension (V = 0.31) but weaker associations with other comorbidities (V range: 0.12–0.18).

Post hoc analyses using Tukey’s HSD test revealed significant differences in metabolic and renal parameters across obesity classes (see Table 5). Blood glucose levels demonstrated the most consistent pattern of increase with obesity severity (*p* < 0.001). The mean difference between Class III and Class I obesity was substantial (25.9 mg/dL, 95% CI: 18.4–33.4, *p* < 0.001), with intermediate but significant differences between Class III and Class II (14.2 mg/dL, 95% CI: 8.7–19.7, *p* < 0.01) and between Class II and Class I (11.7 mg/dL, 95% CI: 6.2–17.2, *p* = 0.01). This stepwise progression suggests a dose-dependent relationship between obesity severity and glycemic dysregulation.

Electrolyte parameters showed significant variations, primarily driven by differences between Class III obesity and lower classes. Potassium levels were significantly higher in Class III than both Class I (mean difference 0.5 mmol/L, 95% CI: 0.2–0.8, *p* < 0.05) and Class II (mean difference 0.3 mmol/L, 95% CI: 0.1–0.5, *p* < 0.05). Similarly, sodium levels were elevated in Class III compared to Class I (mean difference 2.8 mmol/L, 95% CI: 1.4–4.2, *p* < 0.05), though differences between adjacent classes did not reach statistical significance.

Renal function markers demonstrated progressive deterioration with increasing obesity severity. Urea levels showed significant increases across all class comparisons, with the largest difference observed between Class III and Class I (8.3 mg/dL, 95% CI: 5.1–11.5, *p* < 0.001), followed by Class III compared to Class II (5.8 mg/dL, 95% CI: 2.9–8.7, *p* < 0.01). Creatinine levels were particularly elevated in Class III obesity, showing significant differences relative to both Class I (0.4 mg/dL, 95% CI: 0.2–0.6, *p* < 0.01) and Class II (0.3 mg/dL, 95% CI: 0.1–0.5, *p* < 0.05). These findings suggest that the impact of obesity on renal function becomes more pronounced with increasing severity, particularly in Class III obesity.

The analysis of metabolic emergencies demonstrated significant differences between obese and non-obese patients, highlighting the increased risk associated with obesity (Table 6). Hyperglycemia was significantly more prevalent among obese patients (27.9%, n = 45) than non-obese patients (11.0%, n = 30, *p* < 0.001), reflecting the heightened challenges in glycemic control faced by individuals with obesity. Similarly, electrolyte imbalances, including disturbances such as hyperkalemia and hyponatremia, were observed in 23.6% (n = 38) of obese patients, significantly higher than the 9.2% (n = 25) noted in non-obese patients (*p* < 0.001). These findings underscore the profound impact of obesity on fluid and electrolyte homeostasis. AKI was identified in 12.4% (n = 20) of obese patients, nearly double the prevalence found in the non-obese group (5.5%, n = 15, *p* = 0.01), suggesting that obesity exacerbates renal stress and predisposes individuals to kidney-related complications.

Statistical analysis using chi-square tests confirmed the significant differences for all metabolic emergencies between the groups. Hyperglycemia (χ^2^ = 14.76, *p* < 0.001), electrolyte imbalances (χ^2^ = 17.38, *p* < 0.001), and AKI (χ^2^ = 6.63, *p* = 0.01) all showed strong associations with obesity.

A subanalysis comparing obese patients with and without diabetes revealed significantly higher rates of metabolic emergencies in those with diabetes (Table 7). The prevalence of hyperglycemia was more than three times higher in obese patients with diabetes (44.0%) than those without diabetes (14.7%) (*p* < 0.001). Similarly, electrolyte imbalances and acute kidney injury were significantly more frequent in the diabetic subgroup (*p* = 0.03 and *p* = 0.01, respectively). These findings further highlight the critical role of diabetes as a risk amplifier for metabolic emergencies in obese individuals.

## 4. Discussion

The increasing prevalence of obesity worldwide presents substantial challenges to healthcare systems, particularly in emergency and critical care settings [21]. Obesity not only exacerbates the risk of chronic diseases but also predisposes individuals to acute metabolic and renal complications, as highlighted by recent studies [22]. Our findings demonstrate that increasing obesity severity is associated with progressive worsening of metabolic and renal parameters, with Class III obesity representing a distinct high-risk phenotype requiring specialized management approaches.

The findings of this study align closely with recent research highlighting the impact of obesity on metabolic and renal dysfunction. For instance, studies have consistently demonstrated a strong correlation between higher BMI and elevated blood glucose levels, a reflection of worsening insulin resistance and impaired glycemic control in obese patients [23]. The significant elevation in blood glucose levels in Class III obesity (mean difference 25.9 mg/dL compared to Class I, *p* < 0.001) reflects the cumulative impact of adiposity on glucose homeostasis, emphasizing hyperglycemia as a critical risk factor in emergency and critical care settings [24]. These findings are further supported by studies showing that insulin resistance increases proportionally with adiposity, leading to a heightened risk of acute and chronic metabolic complications [23,24].

Obesity-induced metabolic dysfunction, particularly insulin resistance and chronic inflammation, contributes to metabolic derangements observed in our study [25]. Elevated glucose and creatinine levels in Class III obesity align with previous findings linking adipose tissue dysfunction to impaired glucose metabolism and renal stress [26]. Consequently, blood glucose levels rise, as seen in this study, where Class III obesity was associated with the highest glucose levels (*p* < 0.001). Hyperinsulinemia, a compensatory response to insulin resistance, further exacerbates metabolic dysfunction by promoting lipogenesis and impairing lipid metabolism [27]. Obesity is characterized by an imbalance in adipokines—hormones secreted by adipose tissue that regulate metabolic processes. Leptin, which promotes satiety, becomes less effective in obese individuals due to leptin resistance, perpetuating a cycle of overeating and weight gain. Conversely, levels of adiponectin, an anti-inflammatory adipokine, decrease with increasing adiposity. Low adiponectin levels contribute to insulin resistance, increased gluconeogenesis, and lipid accumulation in the liver [28]. These changes promote the development of metabolic syndrome, as reflected in the worsening laboratory parameters observed in our study.

Adipokine dysregulation and oxidative stress contribute to obesity-related metabolic emergencies. Leptin resistance impairs glucose metabolism and cardiovascular function, while reduced adiponectin worsens insulin resistance and inflammation, increasing the risk of diabetic ketoacidosis and acute kidney injury [29]. Excess adiposity also induces oxidative stress, leading to endothelial damage, lipid peroxidation, and systemic inflammation, which exacerbate electrolyte disturbances and cardiovascular instability. Targeting these imbalances may improve metabolic outcomes in high-risk obese patients [30].

The strong association between diabetes and metabolic emergencies (OR: 2.1, 95% CI: 1.4–3.1, *p* < 0.001) compared to hypertension (OR: 1.6, 95% CI: 1.1–2.3, *p* = 0.02) suggests that diabetes exerts a more direct impact on metabolic instability. Chronic hyperglycemia promotes endothelial dysfunction, oxidative stress, and electrolyte imbalances, predisposing patients to hyperglycemic crises and acute kidney injury [31]. Additionally, diabetes-induced autonomic neuropathy may impair compensatory mechanisms during metabolic stress, increasing the likelihood of severe metabolic disturbances. In contrast, while hypertension significantly contributes to renal dysfunction and cardiovascular instability, its acute metabolic impact appears less pronounced in emergency settings [32].

Electrolyte imbalances, particularly elevated potassium and sodium levels, observed in this study are also well documented in the literature. Elevated potassium levels in Class III obesity can be attributed to impaired renal excretion of potassium due to both structural changes in the kidney and functional disruptions caused by hyperinsulinemia [33]. Similar trends have been reported in studies examining the relationship between obesity and hyperkalemia, where Class III obesity amplifies these effects. The observed sodium abnormalities align with findings that obesity-related fluid retention and chronic hypervolemia alter sodium homeostasis, contributing to the development of hypertension and cardiovascular complications [34]. The electrolyte disturbances observed in obesity, including elevated potassium and sodium levels, are influenced by hormonal and renal changes. Hyperinsulinemia increases renal potassium retention by stimulating sodium-potassium ATPase activity, contributing to hyperkalemia. Obesity also alters the renin–angiotensin–aldosterone system, leading to sodium retention and volume expansion [35]. These changes disrupt fluid and electrolyte balance, as evidenced by the significant increases in potassium (*p* = 0.03) and sodium (*p* = 0.02) levels in Class III patients.

Obesity-associated renal dysfunction is multifactorial, with glomerular hyperfiltration and chronic inflammation playing key roles [36]. The progressive increase in creatinine and urea levels in Class III obesity suggests worsening renal function, consistent with obesity-related glomerulopathy [37]. In this study, renal dysfunction showed a clear relationship with obesity severity, as evidenced by progressive increases in both urea and creatinine levels. The significant elevation of these markers in Class III obesity (urea: mean difference 8.3 mg/dL, *p* < 0.001; creatinine: mean difference 0.4 mg/dL, *p* < 0.01, compared to Class I) aligns with the concept of obesity-related glomerulopathy [36,37,38], corroborating the aforementioned well-documented mechanisms and underscoring the deleterious effects of obesity on renal health.

The link between obesity and chronic kidney disease has been extensively studied, with glomerular hyperfiltration serving as a precursor to long-term renal damage. Chronic inflammation, driven by the secretion of pro-inflammatory cytokines from adipose tissue, exacerbates renal injury by promoting fibrosis and oxidative stress. Furthermore, ectopic lipid accumulation in the kidneys, a hallmark of metabolic overload in obesity, disrupts normal renal architecture and function [39]. The progressive increase in renal markers observed in our study, particularly in Class III patients, highlights the cumulative impact of these pathological processes. Elevated levels of urea and creatinine in advanced obesity not only indicate reduced renal function but also point to a distinct clinical phenotype of obesity-related glomerulopathy, as described by D’Agati et al. (2016) [39]. This condition is characterized by structural and functional damage to the kidneys and is increasingly recognized as a major consequence of Class III obesity.

In addition to structural changes, obesity-related renal dysfunction is exacerbated by systemic metabolic derangements. Insulin resistance, commonly observed in obesity, contributes to the activation of the renin–angiotensin–aldosterone system, leading to sodium retention, hypertension, and further renal stress. This mechanistic link between obesity, hypertension, and renal injury highlights the importance of monitoring renal function in obese patients, especially those with advanced obesity grades [40,41]. Our findings of significant renal impairment in Class III patients align with population-based and clinical cohort studies, further validating the relationship between obesity severity and renal dysfunction.

The progression of renal impairment in obesity underscores the need for early detection and intervention. Routine monitoring of renal markers such as urea and creatinine can aid in identifying patients at risk for chronic kidney disease, allowing for timely therapeutic strategies to mitigate renal damage. Additionally, lifestyle modifications, including weight loss and dietary interventions, may help reduce the metabolic and hemodynamic burden on the kidneys, potentially reversing early-stage glomerular changes [42]. These findings emphasize the critical need for a multidisciplinary approach to managing the renal complications associated with Class III obesity.

The stratification of obesity into grades provides a novel framework for understanding the stepwise progression of metabolic and renal dysfunction. This approach aligns with studies emphasizing the importance of BMI categories in predicting clinical outcomes. For example, research by Ortega et al. (2016) demonstrated that obesity phenotypes, including higher BMI categories, are strongly associated with increased metabolic syndrome parameters and cardiovascular risk, findings that parallel the trends observed in our study [43].

In summary, the results of this study are consistent with existing research on the metabolic and renal complications of obesity. However, the significant differences observed across obesity grades, particularly in Class III, provide additional evidence that Class III obesity represents a distinct clinical phenotype requiring targeted interventions. These findings highlight the importance of continued research and tailored management strategies for patients at the highest risk of adverse outcomes.

Although Class III obesity demonstrated large effect sizes for BMI (Cohen’s d = 2.8) and blood glucose (d = 1.2), effect sizes for some electrolyte parameters, such as sodium (d = 0.28), were considerably smaller. While this statistical significance suggests a measurable difference, its clinical impact remains questionable. For example, the observed sodium variation (139.5 mmol/L in Class I vs. 141.3 mmol/L in Class III) falls within the normal physiological range and is unlikely to translate into meaningful clinical consequences unless accompanied by other metabolic imbalances.

This finding underscores the importance of differentiating between statistical and clinical significance, particularly for electrolyte parameters. Mild sodium fluctuations may reflect early dysregulation in fluid homeostasis rather than a direct pathological consequence of obesity. Future research should explore whether these subtle changes contribute to long-term metabolic risk or remain clinically irrelevant in most emergency scenarios [41].

The findings of this study have significant clinical implications. The stratification of obesity into grades provides valuable insights into the progression of metabolic and renal dysfunction, enabling targeted interventions for high-risk patients [44]. Routine monitoring of laboratory parameters such as blood glucose, potassium, sodium, urea, and creatinine can aid in the early detection of complications, reducing the likelihood of critical events [45]. Furthermore, the integration of obesity severity into clinical decision-making may enhance the effectiveness of treatment strategies, particularly in emergency and critical care settings [46].

Emerging treatment options for obesity, including pharmacological interventions such as GLP-1 receptor agonists, have shown promise in reducing metabolic risk factors. Recent evidence from preclinical models [47] suggests that targeting metabolic dysregulation through pharmacotherapy may help prevent severe metabolic emergencies in high-risk obese individuals. These findings highlight the need for further clinical research to evaluate the long-term impact of these treatments on acute metabolic complications. Future studies should explore whether integrating pharmacological interventions with early risk stratification in emergency settings could improve patient outcomes and reduce the burden of obesity-related metabolic crises.

This study has several limitations that should be acknowledged. First, its retrospective design may introduce selection bias and limit causal inferences. Future prospective studies should include a longitudinal component to assess the progression of metabolic emergencies over time. Second, although we adjusted for key confounders such as age and comorbidities, other potential confounders, including medication use and dietary habits, were not accounted for. Future research should incorporate detailed lifestyle and pharmacological data to refine risk models. Lastly, as this is a single-center study, the findings may not be fully generalizable. Multicenter studies with diverse patient populations are needed to validate these findings and establish broader clinical guidelines for the management of obesity-related metabolic emergencies.

Despite these limitations, our study provides valuable insights into the relationship between obesity severity and metabolic emergencies in an emergency department setting. Unlike previous studies, which have primarily focused on obesity’s chronic complications, our research emphasizes its acute metabolic impact. By stratifying patients into different obesity classes and evaluating their associated metabolic alterations, we offer a more detailed understanding of risk stratification for acute metabolic crises. These findings have direct clinical implications, supporting the need for obesity-specific protocols in emergency care and contributing to the growing body of evidence linking obesity severity to acute metabolic dysfunctions. Further prospective research is warranted to confirm these observations and refine early intervention strategies.

Future research should explore the mechanistic pathways linking obesity severity to metabolic and renal dysfunction, including the role of adipokines, chronic inflammation, and oxidative stress. Prospective studies are also needed to evaluate the impact of targeted therapeutic interventions, such as weight loss programs and pharmacological treatments, on laboratory markers and clinical outcomes in obese patients. Additionally, integrating biomarkers of obesity-related complications into clinical practice could improve risk stratification and patient care [48].

Given the increased risk of metabolic emergencies in Class III obesity, we propose a structured emergency department (ED) protocol tailored to this high-risk group. The protocol should include earlier and more frequent monitoring of electrolyte imbalances, particularly sodium and potassium, due to their role in metabolic instability. Weight-based drug dosing adjustments are essential, as pharmacokinetics and volume of distribution are significantly altered in severe obesity. Additionally, enhanced screening for acute kidney injury (AKI) should be implemented using serum creatinine and urine output criteria, given the independent association between Class III obesity and renal dysfunction. A stratified risk assessment for metabolic emergencies should also be incorporated, integrating comorbidities such as diabetes and hypertension into an early warning scoring system. Implementing these targeted protocols in ED settings may improve early detection and intervention, potentially reducing ICU admissions and enhancing patient outcomes.

This study highlights the progressive metabolic changes associated with increasing obesity severity, with significant differences observed in blood glucose, electrolyte levels, and renal function markers across obesity grades. The findings underscore the importance of stratifying obesity by severity to identify high-risk patients and tailor clinical management strategies. Addressing these challenges is important to improving outcomes for obese individuals in emergencies and critical care settings.

## 5. Conclusions

This study underscores the significant role of obesity in increasing the risk and severity of metabolic emergencies, including hyperglycemic crisis, severe electrolyte imbalance, and AKI. The findings highlight that the progression of obesity severity, as stratified by BMI grades, is strongly associated with worsening laboratory parameters such as elevated blood glucose, potassium, sodium, urea, and creatinine levels. Patients with Class III obesity demonstrated the most pronounced metabolic and renal impairments, emphasizing the cumulative burden of Class III obesity. Routine monitoring of laboratory markers, coupled with targeted therapeutic strategies, is critical for mitigating acute health risks and improving outcomes in obese populations. Identifying high-risk patients based on obesity severity can enhance early intervention and optimize resource allocation in EDs. Future research should focus on developing obesity-specific protocols for metabolic emergency management and integrating preventive strategies into healthcare systems to address this growing healthcare challenge.

## Figures and Tables

**Table 1:** Demographic and clinical characteristics of the study population. BMI—body mass index; COPD—chronic obstructive pulmonary disease; SD—standard deviation; %—percentage within the respective group.

Variable	Non-Obese (n = 272)	Obese (n = 161)	Class I (n = 59)	Class II (n = 53)	Class III (n = 49)	*p*-Value	*p*-Value (Obese vs. Non-Obese)	*p*-Value (Class III vs. Class I)	*p*-Value (Class III vs. Class II)	*p*-Value (Class II vs. Class I)
Age (years)	68.1 ± 10.1	72.2 ± 9.4	71.3 ± 9.2	72.5 ± 9.5	73.1 ± 9.6	0.03	0.03	0.002	0.05	0.12
Gender (% female)	131 (48.2)	89 (55.3)	32 (54.2)	29 (54.7)	28 (57.1)	0.08	0.08	0.15	0.20	0.35
BMI (kg/m^2^)	24.5 ± 3.2	36.3 ± 4.7	32.4 ± 1.4	37.2 ± 1.3	43.1 ± 2.8	0.001	0.001	<0.001	<0.01	<0.05
Hypertension (%)	200 (73.5)	146 (90.7)	52 (88.1)	49 (92.5)	45 (91.8)	0.002	0.002	0.001	0.03	0.08
Cardiac Disease (%)	251 (92.3)	144 (89.4)	53 (89.8)	47 (88.7)	44 (89.8)	0.04	0.04	0.22	0.40	0.50
Diabetes Mellitus (%)	32 (11.8)	25 (15.5)	8 (13.6)	9 (17.0)	8 (16.3)	0.05	0.05	0.03	0.08	0.20
Chronic Kidney Disease (%)	145 (53.3)	88 (54.7)	31 (52.5)	29 (54.7)	28 (57.1)	0.45	0.45	0.19	0.25	0.40
COPD (%)	4 (1.5)	2 (1.2)	1 (1.7)	0 (0)	1 (2.0)	0.55	0.55	0.45	0.60	0.75
Neoplasms (%)	54 (19.9)	24 (14.9)	9 (15.3)	8 (15.1)	7 (14.3)	0.12	0.12	0.30	0.35	0.42

Values are presented as mean ± standard deviation; Values are presented as number (percentage); *p*-values compare non-obese vs. all obese groups using Student’s *t*-test for continuous variables and the chi-square test for categorical variables. Statistical significance was set at *p* < 0.05.

**Table 2:** Laboratory findings stratified by obesity grade.

Parameter	Non-Obese (n = 272)	Class I (n = 59)	Class II (n = 53)	Class III (n = 49)	*p*-Value
Glucose (mg/dL) *	127.3 ± 11.2	142.5 ± 12.4	154.2 ± 14.6	168.4 ± 17.5	<0.001
Potassium (mmol/L) *	4.1 ± 0.2	4.3 ± 0.2	4.5 ± 0.3	4.7 ± 0.4	0.03
Sodium (mmol/L) *	138.2 ± 3.4	139.5 ± 3.5	140.1 ± 3.2	141.3 ± 3.1	0.02
Urea (mg/dL) *	41.2 ± 4.8	44.3 ± 5.2	46.8 ± 6.0	50.1 ± 6.7	0.001
Creatinine (mg/dL) *	1.1 ± 0.1	1.3 ± 0.1	1.4 ± 0.2	1.6 ± 0.3	0.002

* Mean ± SD, one-way ANOVA with post hoc Tukey’s test. Normal reference ranges: blood glucose (70–100 mg/dL), potassium (3.5–5.0 mmol/L), sodium (135–145 mmol/L), urea (10–50 mg/dL), and creatinine (0.6–1.2 mg/dL).

**Table 3:** Statistical significance of differences in laboratory markers in obese patients.

Parameter	Correlation Coefficient (r)	95% Confidence Interval	*p*-Value
Glucose	0.52	0.45–0.58	<0.001
Potassium	0.31	0.23–0.38	0.03
Sodium	0.28	0.20–0.35	0.04
Urea	0.43	0.36–0.49	0.001
Creatinine	0.47	0.40–0.53	<0.001

*p*-values were calculated using one-way ANOVA, and a significance threshold of *p* < 0.05 was applied.

**Table 4:** Odds ratios for metabolic emergencies by obesity class.

Variable	Adjusted OR (95% CI)	*p*-Value
Class I Obesity	1.8 (1.2–2.7)	0.008
Class II Obesity	2.4 (1.6–3.6)	<0.001
Class III Obesity	3.2 (2.1–4.9)	<0.001
Age	1.02 (1.01–1.04)	0.03
Hypertension	1.6 (1.1–2.3)	0.02
Diabetes	2.1 (1.4–3.1)	<0.001

Adjusted for age, sex, and comorbidities.

**Table 5:** Post hoc analysis of laboratory parameters across obesity classes.

Blood Parameter	Comparison	Mean Diff.	95% CI of Diff.	Adjusted *p* Value
Glucose (mg/dL)	III vs. I	25.9	18.4 to 33.4	<0.001
	III vs. II	14.2	8.7 to 19.7	<0.01
	II vs. I	11.7	6.2 to 17.2	0.01
Potassium (mmol/L)	III vs. I	0.5	0.2 to 0.8	<0.05
	III vs. II	0.3	0.1 to 0.5	<0.05
	II vs. I	0.2	−0.1 to 0.5	0.24
Sodium (mmol/L)	III vs. I	2.8	1.4 to 4.2	<0.05
	III vs. II	1.3	−0.2 to 2.8	0.09
	II vs. I	1.5	−0.1 to 3.1	0.07
Urea (mg/dL)	III vs. I	8.3	5.1 to 11.5	<0.001
	III vs. II	5.8	2.9 to 8.7	<0.01
	II vs. I	2.5	0.3 to 4.7	<0.05
Creatinine (mg/dL)	III vs. I	0.4	0.2 to 0.6	<0.01
	III vs. II	0.3	0.1 to 0.5	<0.05
	II vs. I	0.1	−0.1 to 0.3	0.32

Results from Tukey’s HSD test following one-way ANOVA; Diff.—differences; 95% CI of Diff.—95% confidence interval of differences.

**Table 6:** Prevalence of metabolic emergencies among obese and non-obese patients.

Metabolic Emergency Type	Obese Patients (n = 161)	Non-Obese Patients (n = 272)	*p*-Value
Hyperglycemia (%)	45 (27.9%)	30 (11.0%)	<0.001
Electrolyte Imbalance (%)	38 (23.6%)	25 (9.2%)	<0.001
AKI (%)	20 (12.4%)	15 (5.5%)	0.01

Hyperglycemia was defined as blood glucose levels exceeding 180 mg/dL, while electrolyte imbalance included cases of hyperkalemia (potassium > 5.0 mmol/L) and hyponatremia (sodium < 135 mmol/L). Acute kidney injury (AKI) was characterized by an increase in serum creatinine of ≥ 0.3 mg/dL within 48 h or a ≥ 50% rise from baseline creatinine. All values are presented as percentages within the respective patient groups.

**Table 7:** Comparison of metabolic emergencies in obese patients with and without diabetes. The prevalence of hyperglycemia, electrolyte imbalances, and acute kidney injury (AKI) was significantly higher in obese patients with diabetes than in those without diabetes. Statistical significance was determined using chi-square tests, with *p*-values indicating the strength of association.

Metabolic Emergency	Obese with Diabetes (n = 25)	Obese Without Diabetes (n = 136)	*p*-Value
Hyperglycemia	11 (44.0%)	20 (14.7%)	<0.001
Electrolyte Imbalance	8 (32.0%)	18 (13.2%)	0.03
Acute Kidney Injury (AKI)	6 (24.0%)	9 (6.6%)	0.01

## Data Availability

Data is contained within the article.
